# Analogical environmental cost assessment of silicon flows used in solar panels by the US and China

**DOI:** 10.1038/s41598-024-60270-9

**Published:** 2024-04-25

**Authors:** Saeed Rahimpour Golroudbary, Mari Lundström, Benjamin P. Wilson

**Affiliations:** Hydrometallurgy and Corrosion, Circular Raw Materials Hub, Department of Chemical and Metallurgical Engineering (CMET), School of Chemical Engineering, Aalto University, PO Box 16200, Espoo, Finland

**Keywords:** Environmental impact, Solar energy

## Abstract

Achieving carbon neutrality requires deployment of large-scale renewable energy technologies like solar photovoltaic (PV) panels. Nevertheless, methods to ascertain the overall environmental impacts PVs and further improve their sustainability are under-investigated. In an effort to provide more understanding of this crucial topic, this research focuses on silicon flows—a key element for manufacturing crystalline silicon PVs. Using system dynamics modeling, we conduct a comprehensive environmental cost assessment of the silicon flows used in PVs based on a comparative analysis between the United States and China as the leading global PV manufacturers. Despite the advancement in wafer quality, material usage reductions and overall price decreases achieved in recent decades, our results project a substantial increase in energy and water consumption in China related to Metallurgical Grade Si (MG-Si), Solar Grade Si (SoG-Si) and cell manufacturing by 2030. An approximate 6.5 times increase of energy and water consumption is observed for c-Si cell manufacturing in China between 2010 and 2020. In 2030, increases of 70% in energy consumption and 69% in water use are estimated for Chinese MG-Si and SoG-Si production. The most significant environmental impact is observed in silicon cell and module manufacturing in both countries, particularly concerning GHG, SO_x_ and NO_x_ emissions. This study provides valuable insights into the environmental impacts of these two major solar panel manufacturing countries by examining the silicon life cycle, from production to end-of-life.

## Introduction

Solar photovoltaic (PV) panels are a vital component of the global transition towards renewable energy sources and the development of PV technologies such as monocrystalline and polycrystalline silicon solar panels currently dominate around 90% of the global PVs market^1^. This increased shift to renewables has increased the consumption of high-purity materials based on silicon (Si), which, even though its crustal abundance of is ~ 295,000 ppm^2^, silica (SiO_2_ with > 99.95% purity) is relatively rare in nature and therefore, it is considered as a critical material by both the European Union and the United States Geological Survey (USGS)^1,3^.

Understanding the spatial variations and temporal changes in greenhouse gas (GHG) emissions during PV production, as well as energy and water consumption through material processing used in PVs, is crucial for successfully realizing global zero-emission goals. The current lack of comprehensive analysis of these factors in literature, poses a significant risk that opportunities in the global PV deployment trajectory that maximize the overall life-cycle environmental benefits of solar power will be overlooked. Addressing this issue is urgent, as it directly impacts the effectiveness of efforts to transition towards a zero-emission future.

Currently, the Chinese and US governments play a significant role in renewable energy industries development as they are seen as key growth sectors, crucial to addressing climate change. According to the IEA Special Report on Solar PV Global Supply Chains^4^, China invested over USD 50 billion in new PV supply capacity between 2011 and 2022—a level of investment that is ten times higher than that spent in Europe over the same period. The US is also accelerating the deployment of PV technologies in line with measures to reduce GHG emissions and it is projected that by 2035, 40% of US electricity demand will be supplied through solar power^5^. Presently, China is responsible for ~ 97% of global silicon wafer production and most of these wafers are shipped from China to be assembled into solar cells. For example, about 75% of the silicon solar cells incorporated into modules installed in the United States are made by Chinese subsidiaries located in just three Southeast Asian countries: Vietnam, Malaysia, and Thailand^6^.

Solar PVs technology is mainly categorized into three generations^7^ and the first comprises both monocrystalline and polycrystalline (multicrystalline) c-Si PV technologies. The next generation includes ‘thin film’ technologies based on amorphous silicon (a-Si) and microcrystalline silicon as well as other semiconductor materials like cadmium–telluride (CdTe), copper–indium–gallium–selenide (CIGS), and gallium–arsenide (GaAs, so-called III–V semiconductor or high concentration PV). Third generation refers to the use of organic compounds (such as dye-sensitized solar cells) that allow low cost-high volume production coupled to flexibility. Nevertheless, the concentration of the crystalline silicon (c-Si) PV supply chain in companies with close ties to China, could pose a significant risk of disruption to the c-Si availability in the US. Whilst the US and China have previously cooperated in renewable energy development, since 2011 the countries have engaged in a protracted and significant trade dispute related to the solar photovoltaics industry^8^.

Although solar PV is considered a low-carbon energy source, its production and deployment can still have ecological consequences if not suitably managed. Consequently, assessment of environmental impacts related to solar PVs can help ensure that the benefits of clean energy generation are not offset by negative effects related to energy and water consumption or pollution. Moreover, evaluation of silicon flows environmental sustainability can help identify opportunities for increased resource efficiency throughout the PV supply chain, which promotes circular economic principles, improved recycling and reduces the need for additional resource extraction. Currently, only a few studies have been conducted on the life cycle assessment of solar panel wastes in China^9^ or the environmental impact of PV compared with other renewable energy sources in the US^10^ and a summary of research related to the environmental impact of c-Si PV throughout its supply chain—with a focus on China and the US—is outlined in Table 1. In addition, this work aims to provide an environmental cost assessment of silicon flows in China and the US with proposals of how the photovoltaic industry can further develop globally as an environmentally friendly technology for electrical energy generation.

**Table 1:** Summary investigation on the environmental impact of crystalline silicon photovoltaic panels in China and the US.

Ref	Objective of study	Method	Supply chain scope				Environmental Impact			Geographical scope
			Mining	Processing	Manufacturing	Recycling	Energy	Water	Emissions	
^11^	Life-cycle energy and environmental performance of PV systems	LCA and net energy analysis	×	×	×		×		×	Regional: Europe, China, USA
^12^	Comparison of CO_2_ emissions of solar PV productions	LCA	×	×	×		×		×	Regional: China, Europe, USA
^13^	Environmental impact of PV system	LCA and sensitivity analysis			×		×		×	China
^14^	Environmental impact of domestic and international trade of raw materials for PV manufacturing	Scenario analysis			×				×	China
^15^	Environmental impact of PV system	LCA: cradle-to-gate approach			×		×		×	China
^16^	Environmental impact of PV system	LCA: cradle-to-gate approach			×		×		×	China
^17^	Environmental impacts of grid-connected power generation in silicon solar modules manufacturing	LCA			×		×		×	China
^18^	Environmental assessment of recycling multi-crystalline silicon photovoltaic panels	LCA				×			×	China

## Methods

### Model description

Several previous studies^19–22^ have reviewed the literature in detail and proposed how emerging LCA-based approaches and simulation modeling would benefit the environmental assessment to support decisions.

A hybrid model for assessing silicon flows used in c-Si PV from mining to manufacturing stages of the supply chain in China and the US has been developed based on system dynamics modeling^23^ combined with a life-cycle assessment (LCA) approach to analyze the structure and behavior of PV systems over time. The causal loop diagram (CLD) of the silicon supply chain is presented in Figure S1 in Supplementary Information (#1). Integrating both methods enables examination of the environmental cost of used silicon flows by considering various feedbacks and loops^24^. GREET model^25^ and Ecoinvent LCA^26^ are used to perform the environmental data for LCA analysis, and Vensim PLE for the stock and flow modeling of materials, energy, water and environmental emissions. The main variables—including flow, stock, and auxiliary variables—are explained through the following mathematical equations and the dynamic model related variables are divided into two groups (endogenous and exogenous) to allow model boundary specification. More specifically, endogenous variables affect and, at the same time, are affected by other system components and parameters, whereas exogenous variables are not directly affected by the system^27^. All environmental parameters and their data^28–32^ (Eqs. 7–12) are derived according to LCA analysis. The life cycle inventory for all parameters is provided in the Supplementary Information (#2). Total silicon production levels were based on the functional unit and system boundary set in this study. Additionally, the direct/indirect energy and water consumptions are calculated according to the production of silicon raw materials and resources for c-Si manufacturing.

The model is composed of three main stages related to silicon wafers and cells manufacture for use in c-Si PVs: (i) Mining; (ii) Processing and (iii) Production. The environmental impacts of each process including energy consumption, water use and associated emissions (CO_2_, CH_4_, SO_x_, NO_x_, CO, VOC, PM2.5, N_2_O, POC, BC, and PM10) are considered in the model. Furthermore, global warming impact based on GHG-100 is also calculated for both countries.

Geographical distribution of silicon flows has been used to simulate the silicon required for PVs technologies from mining to manufacturing, including exports and imports in China and the US. Trade of high-purity silicon (6N-11N) in the US and China was analyzed based on mass flow and shared market value. Normally, the source of silicon is silica—in various natural forms like quartzite—and the silicon required for PV technology is produced by refining Si compounds to a high grade by impurities removal. For example, following mining, silica in quartz sand is reduced in an arc furnace to metallurgical grade Si (MG-Si) followed by purification (6N-9N) to impurities of ≤ 0.01 parts per million by weight (ppmw) for Solar grade Si (SoG-Si)^33^. Generally, this process is achieved through a (modified) Siemens process^34^ and the resultant polycrystalline silicon ingots are sliced into wafers. Additionally, several methods^35,36^ have been investigated for polycrystalline silicon PV cell materials fabrication to increase photoelectric transfer efficiencies and lower production costs, whereas monocrystalline PV cells require another recrystallization known as the intermediate Czochralski (CZ) step^37^. Finally, after wafer slicing, PV cells are encapsulated between glass panes and assembled into a frame.

The stock of silicon ore ($O\left( t \right)$) (endogenous variable) in China and the US is shown in Eq. 1 over the period *“t*_*0*_*-t”*, where *“t*_*0*_*”* is the initial year and *“t”* is the final year. $F_{ij} \left( t \right)$ (endogenous variable) presents the annual production rate of silicon from mining by type *“i”* (with $i$ = *1,2*) including industrial silicon and ferrosilicon and by each country *“j”* (with $j$ = *1,2*) including China and the United States (Eq. 2). $M_{j} \left( t \right)$ (endogenous variable) corresponds to the annual processing of metallurgical grade silicon, which is calculated according to Eq. 3. Historical data for the global mine production of ferrosilicon and silicon are available for the period between 1990 and 2021 from the United States Geological Survey (USGS) data sources^3^.

1 $$ O\left( t \right) = \mathop \smallint \limits_{{t_{0} }}^{t} \left( {\mathop \sum \limits_{j = 1}^{2} \mathop \sum \limits_{i = 1}^{2} F_{ij} \left( t \right) - \mathop \sum \limits_{j = 1}^{2} M_{j} \left( t \right)} \right)dt + O\left( {t_{0} } \right) $$

2 $$ F_{ij} \left( t \right) = \delta_{ij} *O \left( t \right) $$

3 $$ M_{j} \left( t \right) = \alpha_{j} *O \left( t \right) $$

where $\delta_{ij}$ (exogenous variable) is a coefficient of mining silicon in type *“i”* by mining country *“j”* and $\alpha_{j}$(exogenous variable) is a coefficient of processing rate of metallurgical silicon affected by stock of silicon ore in the year *“t”,* $O_{j} \left( t \right)$*.*

Equation 4 estimates the stock of metallurgical grade silicon ($P\left( t \right)$) (endogenous variable) for China and the US over the period *“t*_*0*_*-t”* by a time integral of annual processing of metallurgical grade of silicon $\left( {MG_{j} \left( t \right)} \right)$ (endogenous variable) in each country *“j”* minus processing of produced high purity solar grade of silicon, $g \left( t \right)$ (endogenous variable).

4 $$ P\left( t \right) = \mathop \smallint \limits_{{t_{0} }}^{t} \left( {\mathop \sum \limits_{j = 1}^{2} M_{j} \left( t \right) - g \left( t \right)} \right)dt + P\left( {t_{0} } \right) $$

Equation 5 determines the global stock of silicon used in product *“z”* ($N_{z} \left( t \right)$ which is an endogenous variable) (with $z$ = *1,2,3*) including polycrystalline, monocrystalline and ribbon silicon PV over the period *“t*_*0*_*-t”* by a time integral of ${\text{g}} \left( t \right)$ as the annual rate of silicon solar grade applied in manufacturing minus annual demand of silicon solar grade for manufacturing of product *“z”*, $H_{z} \left( t \right)$ which is an endogenous variable (Eq. 6).

5 $$ N_{z} \left( t \right) = \mathop \smallint \limits_{{t_{0} }}^{t} \left( {g \left( t \right) - H_{z} \left( t \right)} \right)dt + N_{z} \left( {t_{0} } \right) $$

6 $$ H_{z} \left( t \right) = \mu_{z} *D_{z} \left( t \right) $$

where $D_{{\text{z}}} \left( t \right)$ is the demand of global silicon required for product *“z”* in the year *“t”* and $\mu_{z}$ is a share of silicon grade *“k”* in product *“z”*, where both variables are exogenous. Silicon demand can be defined as the aamount of silicon available in country *“j”* and its production sector deficit, which will be supplied by imports.

The equations for environmental assessment of silicon flows used in PV technologies are given in an identical form in the model. In particular, the main processes correspond to i) industrial silicon production, ii) MG-Si production, iii) SoG-polysilicon production, iv) EG-polysilicon production, v) ingot casting of polycrystalline SoG-Si, vi) crystallization and ingot casting for monocrystalline SoG-Si, and vii) wafer cutting of polycrystalline and/or monocrystalline SoG-Si. Primary energy sources through silicon flows include fossil fuel, natural gas, non-fossil fuel, petroleum, nuclear, renewables, coal, and biomass. The energy flows involve exploration, extraction, transportation, processing, and production. Water is also required for all stages, in addition to the generation and transmission of the required energy. Energy consumed in silicon mining is primarily associated with complicated industrial process equipment that requires large amounts of electricity.

Data for silica mining, industrial silicon production and MG-Si can be found in databases such as USGS^3^ and Ecoinvent LCA^26^. SoG-Si production is recognized as a more energy-intensive process than MG-Si due to the purity levels of silicon required for PVs technologies. Data for the LCA of manufacturing processes is available via the GREET model^25^ and other technical reports^38–40^. Table S1 in the supplementary Information (#2) provides details of the dynamic model and data sources used. The global model of the silicon supply chain has been previously outlined in Golroudbary et al.^28^. Accordingly, the “direct” energy (i.e., energy required to process one tonne of silicon at each supply chain stage) and the “indirect” energy (i.e., the upstream energy needed for the fuel and power flows) were considered in the model via Eq. 7, which calculates the total cumulative amount of energy consumed in silicon flows.

7 $$ E_{jr} \left( t \right) = \mathop \smallint \limits_{{{\text{t}}_{0} }}^{{\text{t}}} { }Q_{jr} \left( t \right){\text{dt}} + E_{jr} \left( {t_{0} } \right){ } $$

where $E_{jq} \left( t \right),$ which is an endogenous variable, is the total cumulative amount of energy consumption in country *“j”* through the stage* “r” (*with $r$ = *1,2,3,4),* including mining, MG-Si processing, monocrystalline SoG-Si production, and polycrystalline SoG-Si production in the year *“t”*. $Q_{jr} \left( t \right),$ which is an endogenous variable, represents the annual amount of energy consumed in country *“j”* through the silicon flows in the stage* “r”* in the year *“t”* (Eq. 8).

8 $$ Q_{jr} \left( t \right) = U_{jr} \left( t \right) \times \mathop \sum \limits_{r = 1}^{4} \mathop \sum \limits_{m = 1}^{8} \nu_{rm} $$

where $U_{jr} \left( t \right){ }$(exogenous variable) is the amount of silicon mass in the country *“j”* through the stage* “r”* in the year *“t”*, and $\nu_{rm} { }$ (exogenous variable) is the energy required per one tonne of silicon flow in stage *“r”* mainly from eight energy sources ($m$ = *1,2,…,8),* including fossil fuel, natural gas, non-fossil fuel, petroleum, nuclear, renewables, coal, and biomass.

Water consumption in different silicon supply chain processes was assessed from a “direct” and “indirect” water use perspective. Direct water use is the amount directly linked to the processing production of each separate silicon grade including MG-Si and SoG-Si or the final produced PVs. Indirect water use corresponds to the “hidden” part of the silicon flows in several processes that are directly linked to the energy sources used at the processing location. Equation 9 and Eq. 10 correspond to the total cumulative and annual amount of direct and indirect water consumed for silicon flows for $T_{jr} \left( t \right)$ and $Y_{jr} \left( t \right)$ (which both are endogenous variables), respectively. Where, $\beta_{rl}$ (exogenous variable) corresponds to the intensity of water source *“l”* required per one tonne of silicon flow in stage *“r”,* $l$ = *1,2,…,4* which includes water cooling, water mining, water process, and water reservoir.

9 $$ T_{jr} \left( t \right) = \mathop \smallint \limits_{{{\text{t}}_{0} }}^{{\text{t}}} { }Y_{jr} \left( t \right){\text{dt}} + T_{jr} \left( {t_{0} } \right){ } $$

10 $$ Y_{jr} \left( t \right) = U_{jr} \left( t \right) \times \mathop \sum \limits_{r = 1}^{4} \mathop \sum \limits_{l = 1}^{4} \beta_{rl} $$

GHG emissions and other pollution including global particulate organic carbon (POC), black carbon (BC), nitrous oxide (N_2_O), methane (CH_4_), sulfur oxides (SO_x_), particulate matter with < 2.5 µm (PM2.5), airborne particulate matter with sizes < 10 µm (PM10), nitrogen oxides (NOx), carbon monoxide (CO), volatile organic compounds (VOC), and carbon dioxide (CO_2_), are investigated for all flows within the silicon supply chain in China and the US. Equation 11 and Eq. 12 are used throughout the life cycle stages of the PVs supply chains for the assessment of cumulative and annual emissions related to silicon flows, $V_{jqh} \left( t \right) {\text{and}} X_{jqh} \left( t \right)$(which both are endogenous variables) respectively. IPCC AR5 100-year Global Warming Potential values^41^ of 1 (CO_2_), 36 (CH_4_), and 298 (N_2_O) are used for GHG intensities calculations. $\lambda_{qh}$ (exogenous variable) stands for the annual rate of emission *“p”* (with $p$ = *1,2,…,12*), including GHG, POC, BC, N_2_O, CH_4_, SO_x_, PM2.5, PM10, NOx, CO, VOC, and CO_2_ generated in stage *“r”*.

11 $$ V_{jrp} \left( t \right) = \mathop \smallint \limits_{{{\text{t}}_{0} }}^{{\text{t}}} { }X_{jrp} \left( t \right){\text{dt}} + V_{jrp} \left( {t_{0} } \right){ } $$

12 $$ X_{jrp} \left( t \right) = U_{jr} \left( t \right) \times \lambda_{rp} $$

Extreme tests were conducted in modelling steps for upper and lower limits of exogenous variables to identify the sensitivity of parameters. This aims to clarify the scale of the model and the significance of variables in the defined system boundary.

To assess the validity of model, the outputs are compared with experimental data and their statistical compliance determined. The model was validated using variables like production of silicon metal and ferrosilicon in China, the US and globally. On average, the disparities between the model results and the experimental data for the aforementioned variables were 0.82% and 1.80%, respectively. Equation 13^42^ calculates the replication number of the model. $$N_{m} $$ corresponds to the number of replications, $$S\left( m \right)$$ is data standard deviation, *t* is the test statistic obtained from t-tables, m is the number of initial replications assumed to be 5, α is the confidence interval as 90%, $$\overline{x}\left( m \right) $$ is the data mean, and $$\in$$ is allowable percentage error.

13 $$ N_{m} = \left( {\frac{{S\left( m \right) \times t_{{m - 1,1 - \frac{a}{2}}} }}{{\overline{x}\left( m \right) \in }}} \right)^{2 } $$

## Results

The dynamic model outlined previously allows the complex interactions within the photovoltaic projects in both countries (i.e., China and the US) to be understood. Using the system dynamics method and LCA, this study analyzed the flow of silicon as one of the key elements in solar panels production and related environmental impacts. The proposed model simulates supply chain behavior of silicon used in PVs over time. The environmental costs associated with manufacturing solar panels, including energy and water consumption, greenhouse gas emissions and related pollution, were also analyzed. This holistic approach will allow for the development of more sustainable and environmentally friendly photovoltaic projects, which benefits both countries and the broader global community.

### Comparison of solar PVs installed in the US and China

**Figure 1:**
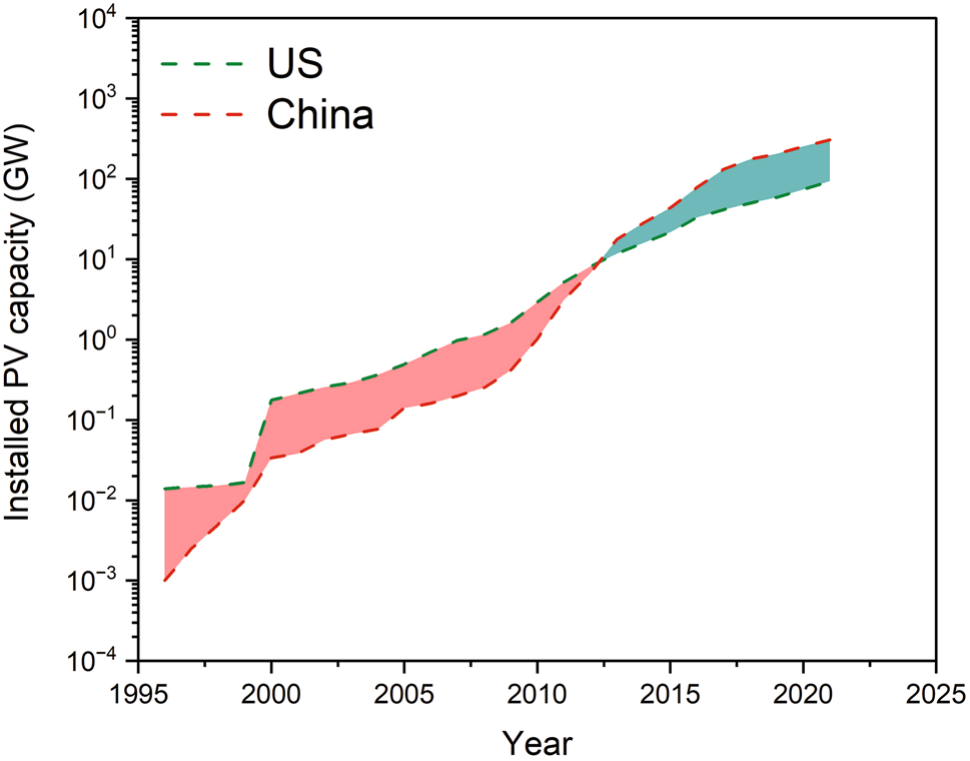
Comparison between installed PV capacities in the US and China (the data retrieved from Our World in Data^44^).

It is important to note that various factors, including policy changes, economic conditions, and technological advancements, can influence the trend of solar PV installations. China and the US have been the world's largest installers of solar PV capacity for several years. Both countries have made significant investments in renewable energy—including solar power—to reduce fossil fuel reliance and combat air pollution. Figure 1 compares installed PV capacities in the US and China between 1996 and 2021 and as can be seen, the annual installations of PV capacity increased significantly in both countries. The US was the global leader in installed PV capacity until around 2013, when China reached parity. By 2021, China's cumulative installed PV capacity surpassed 300 gigawatts, making it the largest solar market in the world with most of the capacity increase attributed to large-scale PV projects^43^. In contrast, the cumulative installed PV capacity in the US exceeded 90 gigawatts in 2021, which was aided by declining solar panel costs, federal tax incentives, state-level policies, and increased public awareness of renewable energy’s benefits. Furthermore, many states have also implemented renewable portfolio standards, requiring a certain percentage of electricity generation from renewable sources, including solar power.

**Figure 2:**
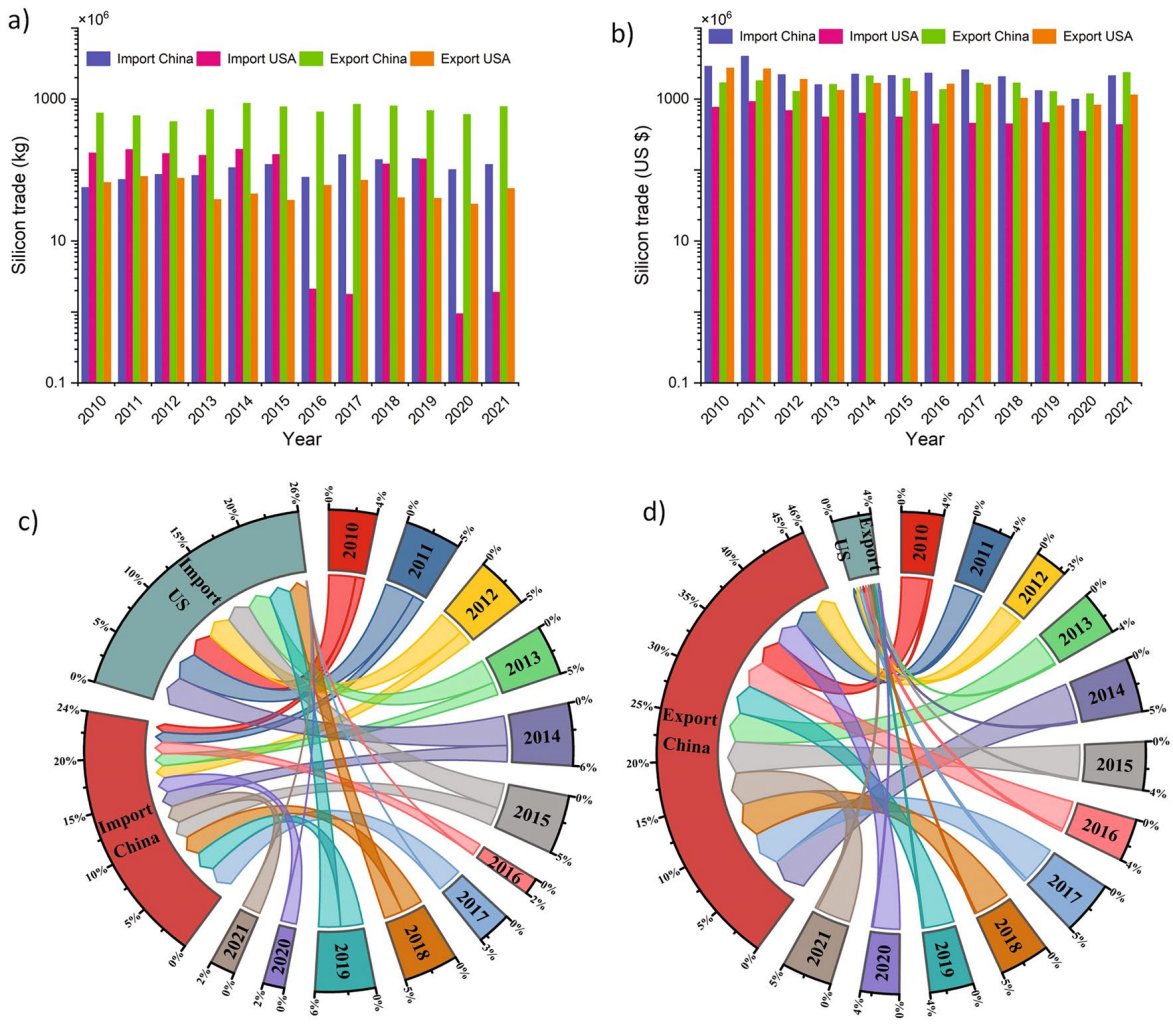
Trade of high-purity silicon (6N-11N) in the US and China: (**a**) Net weight of silicon in kg**;** (**b**) Trade value of silicon in USD; (**c**) Share of the market value of silicon by import in percentage and (**d**) Share of the market value of silicon by export in percentage.

Figure 2 outlines the trade amount and value of high-purity silicon (6N-11N) in the US and China between 2010 and 2021. Detailed information on the quantity and value of exports and imports of high-purity silicon—based on data from 51 exporters and 75 importers in the United Nations Commodity Trade Database (UNCOMTRADE)—shows that in 2021, exports and imports of high-purity silicon reached around 200 thousand tonnes (kt) with a value of USD 4.4 billion and 190 kt (USD 4.2 billion), respectively. China has been a significant player in the production and export of high-purity silicon due to its significant polysilicon industry, with numerous manufacturers supplying both the domestic and global industrial solar and semiconductor markets.

On the other hand, the US has also been a consumer of high-purity silicon, primarily for its domestic solar and semiconductor manufacturing sectors. While the US has some polysilicon production capacity, it has historically imported a significant portion of its requirements. Various factors, including market demand, trade policies, and geopolitical dynamics, have influenced the trade of high-purity silicon between the US and China. Tariffs and trade disputes between the two countries have impacted the flow of polysilicon trade. For example, in recent years, trade tensions and tariffs have been imposed by both countries on various products, including solar-related materials^45^. Such trade measures can affect the trade volumes and prices of high-purity silicon between the US and China with analysis indicating that China increased high-purity silicon imports by 111% in 2021 compared with 2010, whereas exports rose around 24% increase over the same period. In contrast, the US significantly decreased trade, with around 98% of imports and 18% of exports of high-purity silicon between 2010 and 2021.

### Comparison of environmental impacts of silicon flows

**Figure 3:**
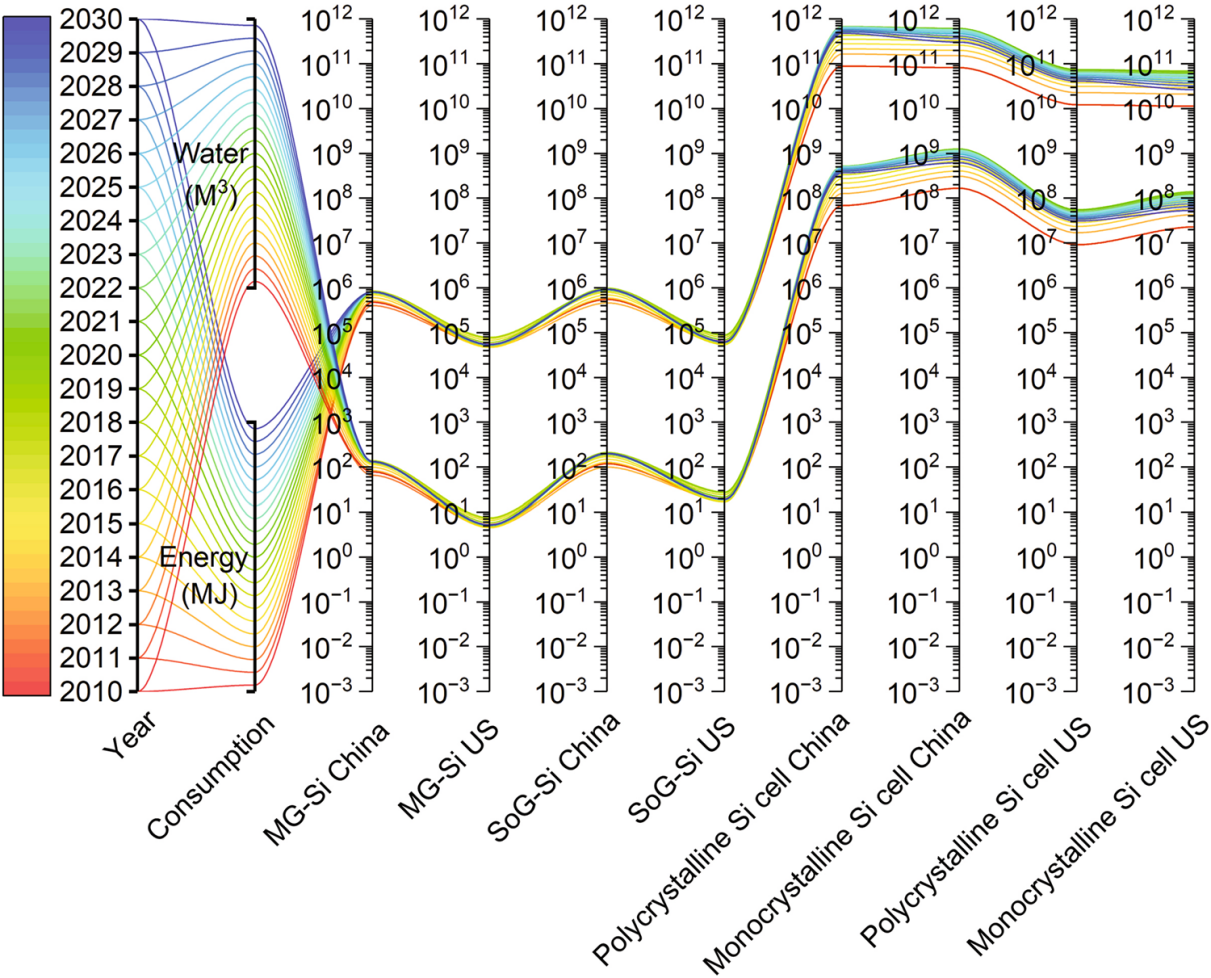
Energy consumption, in megajoules (MJ) and water consumption, in cubic meters, through silicon mining, processing (including metallurgical grade silicon (MG-Si) and solar grade silicon (SoG-Si) production) and photovoltaic cell manufacturing in the US and China. Data are available in Supplementary Information (#3).

Silicon mining, processing and solar PV manufacturing all require significant levels of energy and water Consequently, the proposed model assessed the related consumption for silicon flows in both countries and determined that the figures for China are higher than in the US (Fig. 3). The annual comparison of each stage shows around 63% and 64% increase in China of energy and water consumption for MG-Si and SoG-Si production between 2010 and 2020, with further increases to 70% and 69% respectively estimated by 2030. The results also indicate an approximate 6.5 times increase in energy and water consumption for c-Si cell manufacturing in China between 2010 and 2020. On the other hand, the same estimates show that the energy and water consumption growth for MG-Si and SoG-Si production in the US is only around 58% and 54%, respectively.

Globally, the highest consumers of energy and water for silicon mining and processing stages in 2010 were China (61%) followed by the US (9%). The detailed calculations show that energy consumption for MG-Si production will reach 0.8 million MJ by China and 0.05 million MJ by the US in 2030, whereas energy consumption for SoG-Si production is expected to reach 0.9 million MJ (China) and 0.06 million MJ (US). In the manufacturing sector, it is estimated that 478 GJ and 304 GJ of energy for polycrystalline and monocrystalline Si cell production will be consumed by China in 2030, while these same figures will only be around 4 GJ and 3 GJ respectively, in the US.

The water consumption assessment estimates that China will need almost 335 cubic meters (m^3^) of water for MG-Si (~ 133) and SoG-Si (~ 202) production in 2030, whilst the US estimates are only around 5 m^3^ and 19 m^3^. In the case of the manufacturing sector, these predictions are higher at 369 and 620 million m^3^ of water consumption (China) for polycrystalline and monocrystalline Si cell production *cf.* 30 and 54 million m^3^ of water in the US. This finding helps identify areas for improvement and promote the development of more sustainable energy and water consumption practices throughout the solar PV industry.

**Figure 4:**
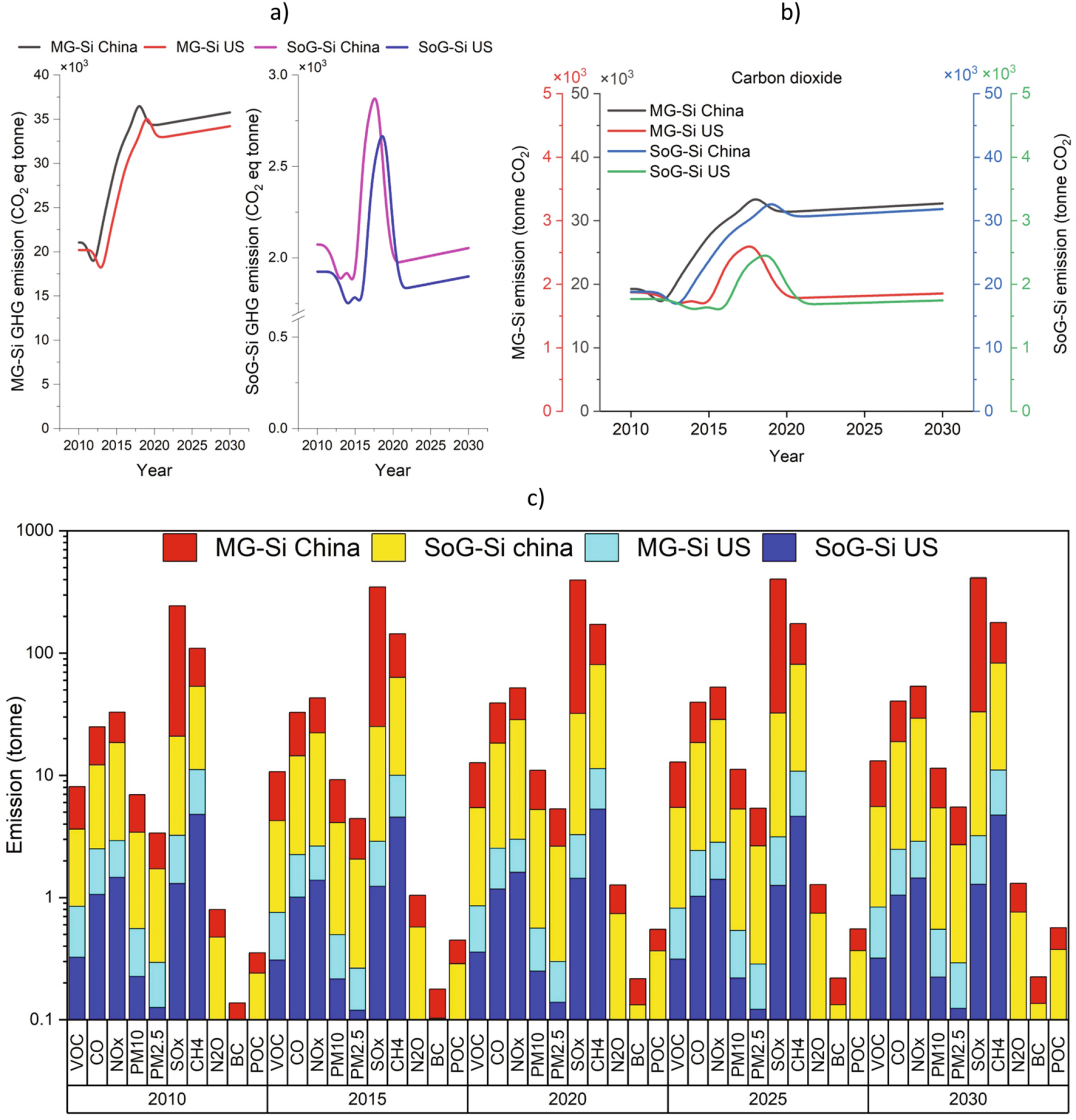
Environmental impact of metallurgical and solar grade of silicon production in the US and China between 2010 and 2030. (**a**) greenhouse gas emissions in tonnes**;** (**b**) carbon dioxide (CO_2_) emission in tonnes; (**c**) other pollution in tonnes. Pollution includes Particulate organic carbon (POC), black carbon (BC), nitrous oxide (N_2_O), methane (CH_4_), sulfur oxides (SO_x_), particulate matter with sizes smaller than 2.5 µm (PM2.5), airborne particulate matter with sizes smaller than 10 µm (PM10), nitrogen oxides (NO_x_), carbon monoxide (CO), and volatile organic compounds (VOC). Data are available in Supplementary Information (#4).

Although the emissions associated with solar PV production are significantly lower compared to those from fossil fuel-based energy generation over the life cycle of the solar panels, an assessment of the emissions generated by the proliferation of solar PV technologies is essential to ensure their full life cycle sustainability. Three main categories can be considered for emissions generated by the primary production of metals including: direct process-related emissions, auxiliary processes emissions and indirect emissions mainly due to the electrical energy used in the production process. Solar PV production can result in greenhouse gas and other emissions, mainly from the energy-intensive processes involved in silica mining, silicon processing and purification, wafer production, cell, and module manufacturing. Generally, the indirect emissions from the electrical energy production contribute a significant part of the overall specific carbon footprint per kg of silicon produced. The trends of annual carbon dioxide and GHG emissions generated by MG-Si and SoG-Si production and other related pollutions (POC, BC, N_2_O, CH_4_, SO_x_, PM2.5, PM10, NO_x_, VOC, and CO_2_) between 2010 and 2030 are shown in Fig. 4. It is worth noting that advancements in manufacturing processes, and supply chain sustainability are continuously being pursued to reduce the negative impact of solar PV production^46^, however, an improvement in technologies from an environmental perspective is still needed for production processes. For example, the environmental impact of quartz mining and silica sand extraction for PV has not been updated for over 15 years, therefore the environmental impact PV production may be underestimated.

The GHG emitted through MG-Si production is estimated to increase from around 21.1 kt CO_2_eq to 35.7 and from 20.2 CO_2_eq kt to 34.2 between 2010 and 2030 in China and the US, respectively. These results also indicate a decrease in carbon footprint of SOG-Si production after 2018 due to the reduced share of silicon applications in high technology. Although SOG-Si production through the metallurgical route seems promising from a cost point of view, for purity reasons, it is still struggling to be accepted by solar cell makers. In addition, several mergers related to SOG-Si production that have occurred during the last decade that have resulted in a consolidation, so that now only a few companies remain in the market. Nevertheless, advanced research and development efforts are ongoing to further improve silicon material and cell manufacturing technologies (i.e., decrease in wafer thickness and silicon consumption over time). Due to such prevailing market conditions, the utilized model assumes crystalline silicon will remain the dominant technology for solar cells by 2030.

Amongst the different pollution categories specified, CO_2_, SO_x_ and NO_x_ emissions were found to be the highest. It is estimated that the amount of CO_2_ emissions attributable to MG-Si and SOG-Si production in China will reach around 32.7 kt and 31.8 kt in 2030, whereas over the same period, the estimated CO_2_ emissions due to MG-Si and SoG-Si in the US will reach 1.9 kt and 1.7 kt. The MG-Si production SO_x_ and NO_x_ emission levels are predicted to be approximately 380 t and 25 t (China) and around 2 t SO_x_ and 1.4 t NO_x_ (US) by 2030. Similar estimates for these pollutant emissions are also predicted for the SOG-Si production in both nations.

**Figure 5:**
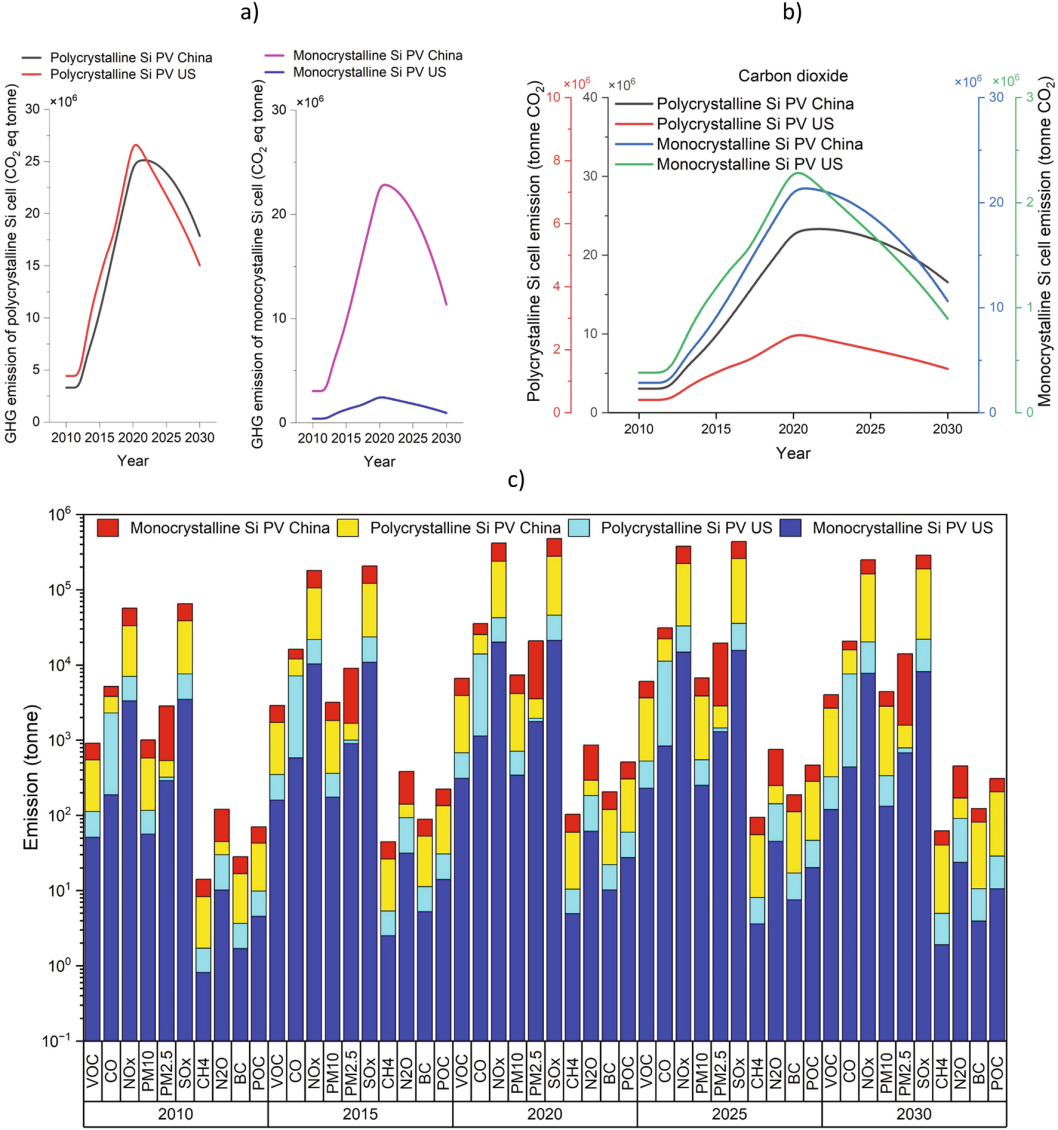
Environmental impact of manufacturing photovoltaic cells in the US and China between 2010 and 2030. (**a**) greenhouse gas emissions in tonnes; (**b**) carbon dioxide emission in tonnes and (**c**) other pollution in tonnes. Data are available in Supplementary Information (#5).

Figure 5 shows the environmental impact of polycrystalline and monocrystalline silicon cell manufacturing in the US and China. It is notable that the amount of environmental impact in the manufacturing stage is higher than in the processing stage. The highest pollution in PV manufacturing corresponds to SO_x_, NO_x_, followed by PM 2.5 and CO. Also, it is estimated that there will be around 18 Mt CO_2_eq and 11 Mt CO_2_eq of GHG emissions due to polycrystalline and monocrystalline silicon cell manufacturing, respectively, in China by 2030 compared with the US, with 15 Mt CO_2_eq and 1 Mt CO_2_eq. The detailed calculations show around 2.7–4.4 and 1.3–2.4 times increase in 2030 compared with 2010 estimated in the amount of generated pollution through polycrystalline and monocrystalline silicon PV production in China and the US, respectively. It should be noted that the trend of emissions is mainly affected by changes in production volume and supply of electrical energy generated from fossil fuels or low-carbon energy resources such as renewable sources. Therefore, these findings emphasize the importance of balancing the consumption of critical materials and promotion of a sustainable green economy transition through the appropriate allocation of flows of different silicon grades, which can lead to energy, water, and GHG emissions savings as proposed previously^28^.

### Comparison of environmental costs of silicon flows

The environmental costs associated with silicon flows used in solar PV manufacturing include factors such as energy consumption, water usage, emissions of greenhouse gases and other pollutants, as well as the impact on local ecosystems and communities. These costs are incurred at various stages of the manufacturing process, from the extraction of raw materials to the production of silicon wafers and solar cells. Carbon price is calculated based on the supply and demand for carbon credits within an economy. In this case, the price of carbon is set by the government, and the market determines the level of emission reductions incentivized by the price—both the US and China have a carbon emission trading system. The US mainly produces polysilicon, but it is exported to manufacture cells and panels in other countries. Chinese production has increased for each manufacturing stage, e.g., for polysilicon (30%)^47^.

**Figure 6:**
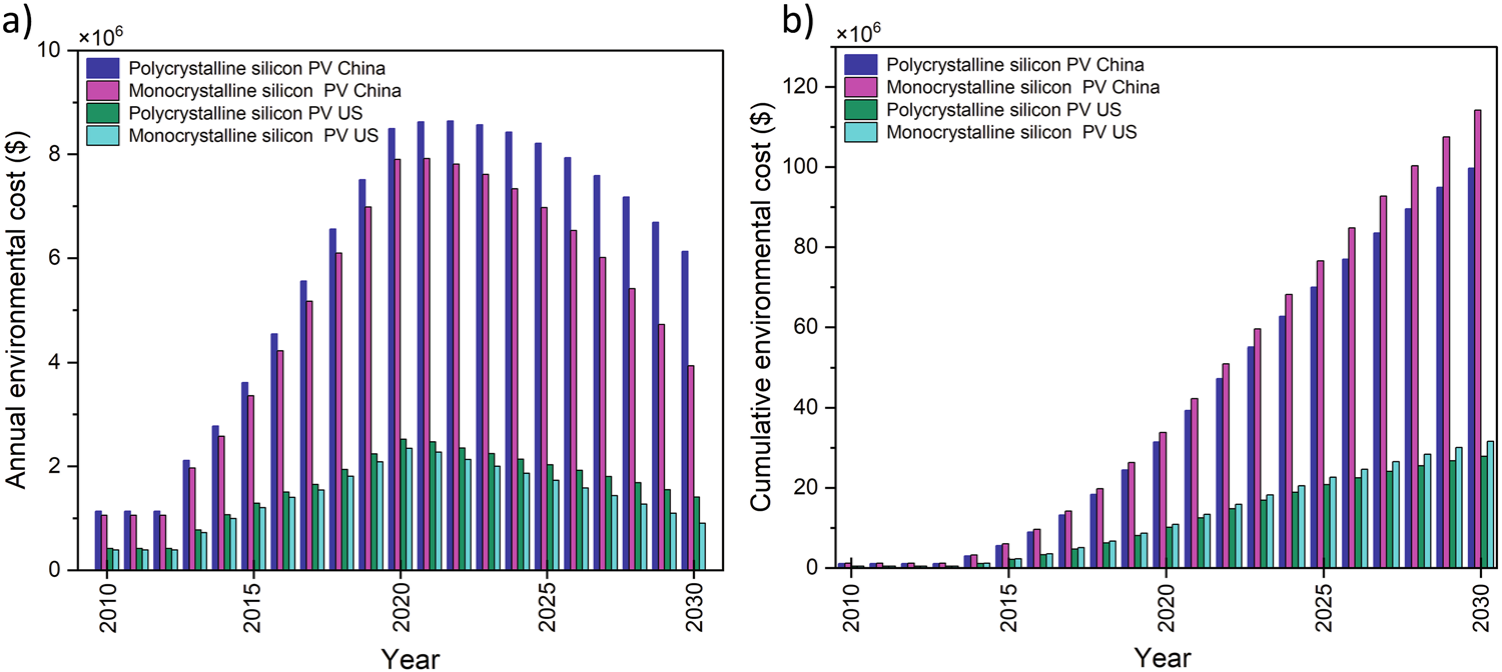
Environmental cost of using silicon for producing photovoltaic cells in the US and China. Data are available in Supplementary Information (#6).

The results, shown in Fig. 6, indicate the annual reduction of environmental cost for silicon PV manufacturing in both countries after 2021. This reduction is mainly influenced by increased efficiency as well as reductions in material and electricity consumption. The material intensity of silicon in c-Si PV shows a notable drop and a more detailed analysis estimates that the silicon intensity in solar PV panels will decrease from 1.1805 (kg/panel) to 1.0732 between 2020 and 2030. Furthermore, historical data provided by the Fraunhofer Institute for Solar Energy Systems (ISE) shows silicon usage in c-Si PVs has decreased from 16 g/W in 2004 to 4 g/W in 2018. Nevertheless, when the systematic effect of other factors such as the demand growth of technology deployment of PVs and delay mechanisms in the system are considered, the annual environmental cost has increased over the same period. The cumulative environmental cost of manufacturing for 20 years (2010–2030) is estimated around USD 0.09 billion (polycrystalline, China) and USD 0.11 billion (monocrystalline, China), whilst in the US the estimates are USD 0.12 billion for (polycrystalline) and USD 0.03 billion (monocrystalline).

## Discussion

Silicon PVs deployment has moved at a remarkably fast pace alongside steady efficiency gains at the cell and module level for commercial products. In addition, many current studies are attempting to further improve silicon material and decrease its cost, as well as to improve cell manufacturing for the realization of next generation products that incorporate passivating contacts. Nonetheless, assessment of environmental impact of production processes through the PV technology supply chain is essential to ensure its sustainability and this work outlines the environmental cost of solar PV supply chain for the US and China as leading global PV manufacturers with significant local reserves of silicon. This study introduced a dynamic model including life cycle assessment and system dynamics modeling to examine the current and future environmental situation of development of PV technology by considering various feedback loops. However, major gaps in the literature can misrepresent a holistic view of material flows used in renewable technologies such as solar PV, especially with respect to the environmental costs of its raw materials like silicon.

Based on our quantitative analysis, a significant increase was observed in energy and water consumption by China related to the production of metallurgical and solar grades of silicon and PV cell manufacturing by 2030. In addition, there is also an observable an increase in environmental emissions related to the different stages of the Chinese supply chain, especially for silicon cell and module manufacturing. However, from a holistic perspective, by projecting annual reductions in environmental costs after 2021, the study highlights the potential for increased efficiency and reductions in material consumption to positively influence the environmental impact of manufacturing processes. Additionally, the analysis of historical data and estimations for future trends in silicon intensity in solar PV panels provides valuable information for industry stakeholders and policymakers aiming to track and manage the environmental impact of solar PV manufacturing over time. This analysis indicates how changes in manufacturing demand affect the environmental consequences associated with deploying solar PVs. Reductions in material and energy consumption would significantly influence annual environmental costs for silicon PV manufacturing. Industries with high international trading intensity and where emission costs are a significant fraction of revenue are considered to be at risk of carbon leakage—silicon production scores very high in both of these dimensions. Additionally, location has a significant impact on the overall carbon footprint of PV manufacturing. The PV supply chain is currently located in regions where the decarbonization of the electrical grid is expected to be slower than other regions, especially in the US as shown in more detail within the 2017 National Renewable Energy Laboratory (NREL) technical report^47^.

The findings of this study are essential to help us understand how China and the US can reduce their impacts through the development of more sustainable pathways for the renewable energy transition as the main global producers of such technologies. The analogical environmental cost assessment provided in this study can be harmonized with technological advancements and increased market penetration of solar power as a renewable energy source. Consequently, this study has important implications for other regions or industries as they look to secure a reliable supply of green energy.

### Supplementary Information

Supplementary Information 1.

Supplementary Information 2.

Supplementary Information 3.

## Data Availability

The authors declare that the data supporting the findings of this study are available within the article and its Supplementary Information files.
