# Supplementary material for: Analogical environmental cost assessment of silicon flows used in solar panels by the US and China
Source: Sci Rep. 2024 Apr 25;14:9538. doi: 10.1038/s41598-024-60270-9 (PMC11045744; doi:10.1038/s41598-024-60270-9)
Supplement: Supplementary file 1 — Supplementary Information 1. [file 41598_2024_60270_MOESM1_ESM.docx]

**Description of Additional Supplementary Files**

**File Name:** Supplementary Information

**Description:** The Supplementary Information includes: Energy consumption, in megajoules (MJ) and water consumption, in cubic meters, through silicon mining, processing (including metallurgical grade silicon (MG-Si) and solar grade silicon (SoG-Si) production) and photovoltaic cell manufacturing in the US and China; Environmental impact of metallurgical and solar grade of silicon production in the US and China; Environmental impact of manufacturing photovoltaic cells in the US and China; Cumulative environmental cost of using silicon for producing photovoltaic cells in US and China ($); Table S1. Data sources for all parameters of the model of silicon supply chain; Figure S1: Causal Loop Diagram (CLD) of Silicon Supply Chain.
