# Supplementary figures and images for: Analogical environmental cost assessment of silicon flows used in solar panels by the US and China
Source: Sci Rep. 2024 Apr 25;14:9538. doi: 10.1038/s41598-024-60270-9 (PMC11045744; doi:10.1038/s41598-024-60270-9)

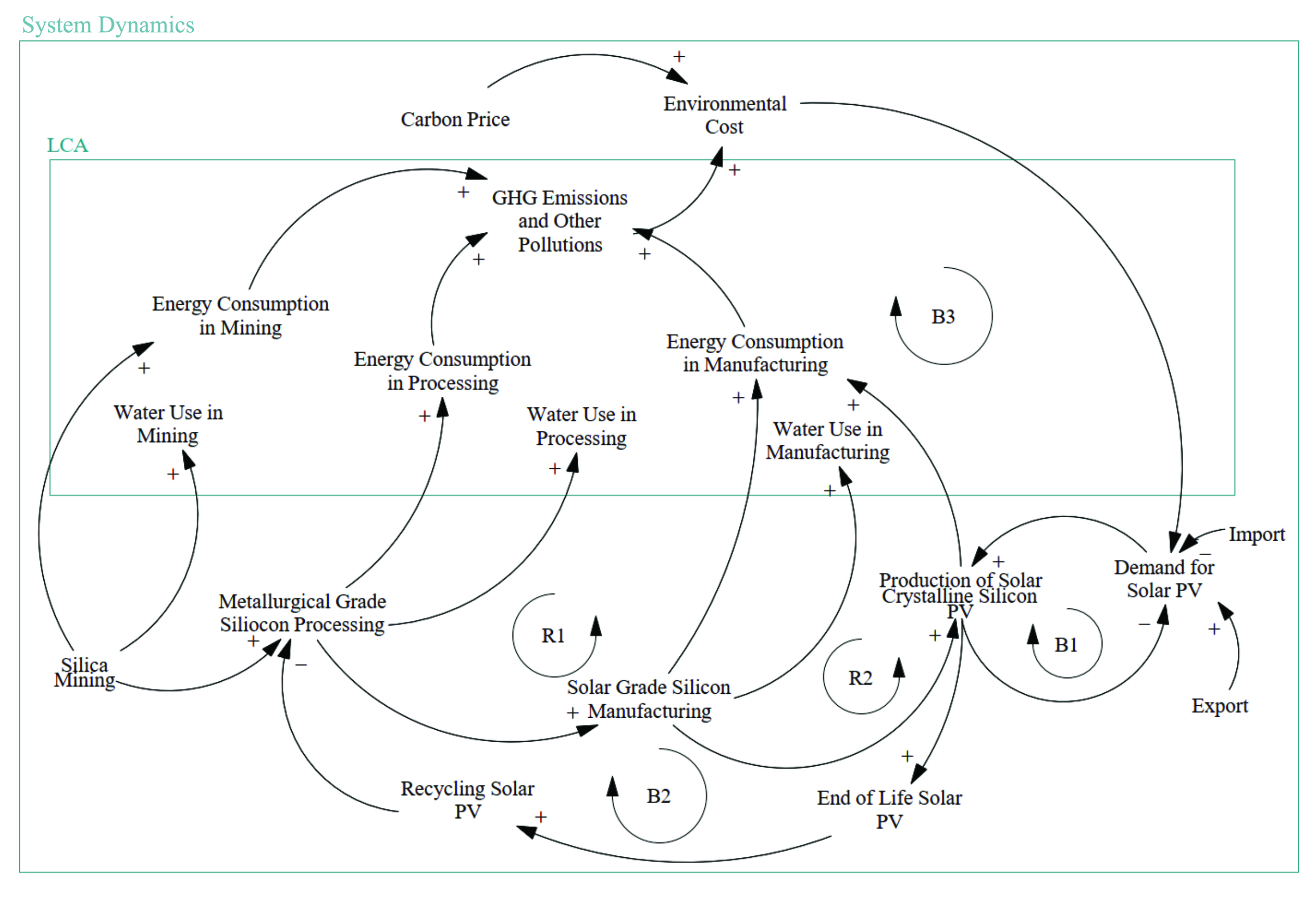

Supplement: Supplementary file 2 — Supplementary Information 2. [file 41598_2024_60270_MOESM2_ESM.jpg]
